# Inter­molecular inter­actions in a phenol-substituted benzimidazole

**DOI:** 10.1107/S2056989019001270

**Published:** 2019-01-29

**Authors:** David K. Geiger, H. Cristina Geiger, Shawn M. Moore

**Affiliations:** a Department of Chemistry, SUNY-College at Geneseo, Geneseo, NY 14454, USA

**Keywords:** crystal structure, hydrogen bonds, C—H⋯π inter­actions, Hirshfeld surface, density functional theory, inter­action energy, benzimidazole

## Abstract

Hydrogen bonding plays an important role in the design of solid-state structures and gels with desirable properties. 1-(4-Hydroxybenzyl)-2-(4-hydroxyphenyl)-5,6-dimethyl-1*H*-benzimidazole was isolated as the acetone disolvate, C_22_H_20_N_2_O_2_·2C_3_H_6_O. O—H⋯N hydrogen bonding between benz­imidazole mol­ecules results in chains parallel to [010]. One of the acetone solvate mol­ecules participates in O—H⋯O hydrogen bonding with the benzimidazole derivative. C—H⋯π inter­actions are observed in the extended structure. Hirshfeld surface analysis was used to explore the inter­molecular inter­actions and density functional theory was used to estimate the strength of the hydrogen bonds.

## Abstract 2

The solid-state structure of 2-(4-hy­droxy­phen­yl)-1-[(4-hy­droxy­phen­yl)meth­yl]-5,6-dimethyl-1*H*-benzimidazole acetone disolvate exhibits O—H⋯N hydrogen bonds between benzimidazole units and O—H⋯O hydrogen bonds with one of the acetone solvate mol­ecules as the acceptor. Density functional theory is used to estimate the strength of the inter­actions.

## Chemical context   

The formation of a gel rather than a crystalline solid depends on the ability of the dissolved gelator to self-assemble into a three-dimensional network structure incorporating the solvent *via* non-covalent inter­actions rather than self-assembly followed by crystallization. The study of the gelation properties of small organic compounds (organogelators) is of importance in soft-matter research because of possible biomedical applications (Lau & Kiick, 2015 ▸; Huynh *et al.*, 2011 ▸; Ye *et al.*, 2014 ▸), including potential use in tissue engin­eering (Xavier *et al.*, 2015 ▸; Yan *et al.*, 2015 ▸), drug delivery and diagnostics (Wu & Wang, 2016 ▸; Tibbitt *et al.*, 2016 ▸), and medical implants (Liow *et al.*, 2016 ▸; Yasmeen *et al.*, 2014 ▸).

Our efforts in this area include the preparation, structural characterization and exploration of the inter­molecular inter­actions in long-chain ester-substituted biphenyl derivatives (Geiger, Geiger, Moore *et al.*, 2017 ▸; Geiger, Geiger, Roberts *et al.*, 2018 ▸) and phenyphenol derivatives (Geiger, Geiger & Morell, 2018 ▸). We have also reported a novel long-chain ester-substituted benzimidazole gelator (Geiger, Zick *et al.*, 2017 ▸).
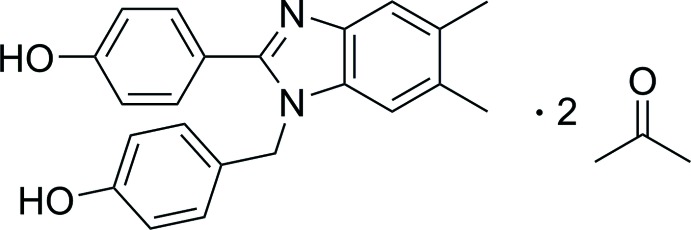


In our continuing efforts to exploit benzimidazole as a gelator core, we synthesized 1-(4-hydroxybenzyl)-2-(4-hy­droxy­phenyl)-5,6-dimethyl-1*H*-benzimidazole in the hope of using it as a starting material to prepare derivatives with a propensity for gelation. This compound was isolated as the di-acetone solvate, (1), and we report herein its structural characterization and an exploration of its three-dimensional superstructure, including an examination of hydrogen-bond strengths.

## Structural commentary   

**Figure 1:**
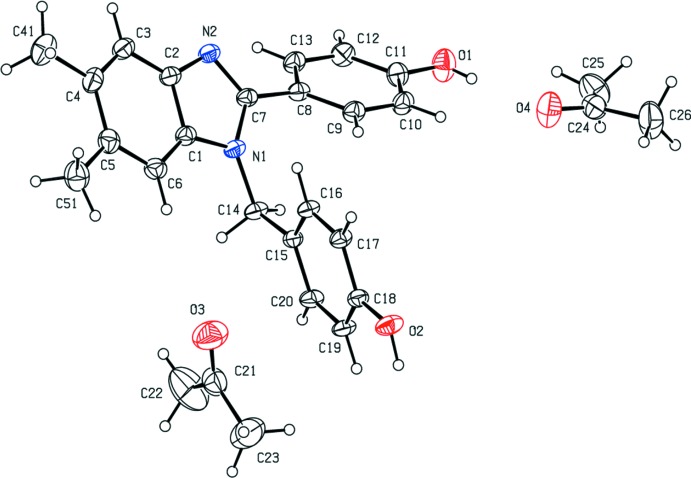
View of the mol­ecular structure of (1) showing the atom-labeling scheme. Displacement ellipsoids for non-hydrogen atoms are drawn at the 30% probability level.

A view of the mol­ecular structure of (1) with the atom-labeling scheme employed is seen in Fig. 1 ▸. The bond lengths and angles are all within the range reported for similar disubstituted benzimidazole derivatives (*c.f.* Geiger & DeStefano, 2016 ▸). The benzimidazole moiety is planar with the largest deviation for C7 [0.0344 (13) Å]. The 2-(4-hy­droxy­phen­yl) substituent is canted at an angle of 44.18 (7)° from the benzimidazole plane and the N2—C7—C8—C13 torsion angle is −43.7 (2)°.

In addition to the benzimidazole, the asymmetric unit of (1) contains two acetone mol­ecules, one of which uses its carbonyl oxygen atom as acceptor in an O—H⋯O hydrogen bond (see Table 1 ▸). The hydrogen-bonded acetone mol­ecule exhibits a slightly longer C—O bond distance than the other acetone mol­ecule [1.212 (3) Å *versus* 1.192 (3) Å]. This observation is consistent with previous results (Ichikawa, 1979 ▸).

## Supra­molecular features   

**Figure 2:**
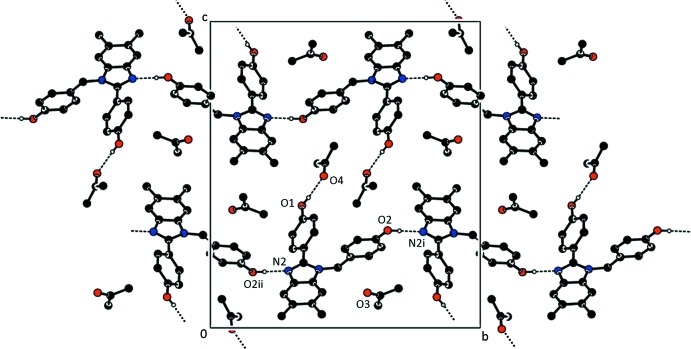
Partial packing diagram of (1) showing the O—H⋯N hydrogen bonding resulting in chains along [010]. Only H atoms involved in the inter­actions are shown. Symmetry codes: (i) −*x* + 2, *y* + 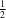, −*z* + 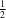; (ii) −*x* + 2, *y* − 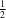, −*z* + 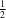.

Fig. 2 ▸ and Table 1 ▸ show the hydrogen-bonding network exhibited by (1). Each of the phenol groups behaves as a donor in a hydrogen bond. The 2-(4-hy­droxy­phen­yl) substit­uent participates in an O—H⋯O inter­action with one of the acetone solvate mol­ecules as the acceptor. The 1-(4-hy­droxy­phen­yl)methyl substituent forms an O—H⋯N hydrogen bond in which an adjacent benzimidazole moiety serves as the acceptor. The result is a chain structure that runs parallel to [010].

**Figure 3:**
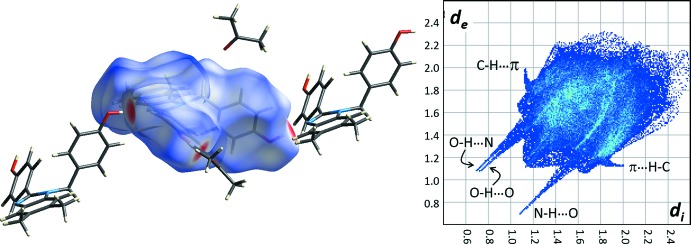
Hirshfeld surface (left) and fingerprint plot (right) for the benzimidazole moiety of (1).

**Figure 4:**
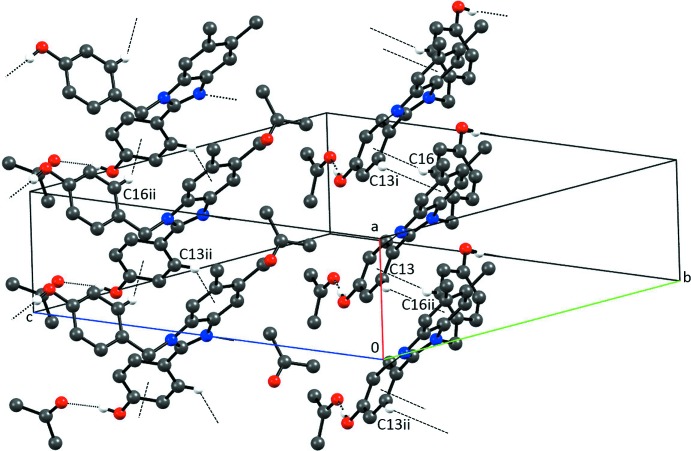
Partial packing diagram of (1) showing the chains along [100] resulting from C—H⋯π inter­actions. Only H atoms involved in inter­actions are shown. Symmetry codes: (i) *x* + 1, *y*, *z*; (ii) *x* − 1, *y*, *z*; (iii) *x*, −*y* + 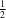, *z* + 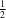.

Fig. 3 ▸ shows the Hirshfeld surface and fingerprint plot for the disubstituted benzimidazole moiety. The prinicpal hydrogen-bonding inter­actions are clearly visible. The surface coverages corresponding to H⋯O and H⋯N inter­actions are 16.0% and 5.9%, respectively. There are no significant π–π inter­actions observed. The fingerprint plot does, however, reveal a weak C—H⋯π inter­action that involves C16—H16 with the 2-(4-hy­droxy­phen­yl) substituent ring system and C13—H13 with the benzene ring of the benzimidazole moiety (see Table 1 ▸ and Fig. 4 ▸). These inter­actions are between mol­ecules translated along the *a* axis. The surface coverage corresponding to H⋯C inter­actions is 24.7%.

The inter­action energies were calculated using density functional theory with the CE-B3LYP/6-31G(*d,p*) functional/basis set combination (see Section 7 for details). The results of the calculations are reported in Table 2 ▸. As expected, the electrostatic component is the primary contributor to the traditional hydrogen-bonding inter­actions and the dispersive component dominates for the C—H⋯π inter­actions. The C—H⋯π inter­actions appear to reinforce each other with the sum of their contributions exceeding that of the traditional hydrogen-bond energies.

The M06 suite of density functionals are reported to outperform B3LYP for dispersion and ionic hydrogen-bonding inter­actions (Walker *et al.*, 2013 ▸; Zhao & Truhlar, 2008 ▸) and so the M06-2X/6-31G(*d,p*) functional/basis set combination was also used to calculate the inter­action energies. The results are found in Table 2 ▸. The value obtained using the M06-2X functional compares favorably with the CE-B3LYP functional result for the phenol⋯acetone hydrogen bond, but the values are decidedly less for the C—H⋯π and the inter-benzimidazole O—H⋯N hydrogen bonds. The calculations employing the M06-2X functional were performed in the gas phase; however, in the solid state, inter­molecular inter­actions do not occur in isolation, which may account for the difference in results.

O—H⋯O hydrogen bonds exhibit a large range of energies. For example, reported *o*-hydry­oxy ketones have intra­molecular hydrogen-bond strengths of −26.8 to −54.8 kJ mol^−1^ (Rusiniska-Roszak, 2017 ▸) and a series of CX[4} and CX[5] calixarenes have calculated O—H⋯O energies ranging from −19.2 to −34.4 kJ mol^−1^ (Khedkar *et al.*, 2012 ▸). The O—H⋯O hydrogen-bond energies in cyclo­dextrin conformers were found to range from −4.6 to −34.7 kJ mol^−1^ (Deshmukh *et al.*, 2011 ▸). As a final example, the hydrogen-bond energy in the optimized water dimer is −21 kJ mol^−1^ and in the hexa­mer water cluster it is −42 kJ mol^−1^ (Wendler *et al.*, 2010 ▸). The value obtained in the present study (Table 2 ▸) is comparable to these values.

The O—H⋯N hydrogen-bond strength is greater than the O—H⋯O hydrogen-bond strength in this example (Table 2 ▸), as has been observed for H_3_SiOH with O- and N-atom acceptors (Beckmann & Grabowsky, 2007 ▸). For comparison, the intra­molecular O—H⋯N hydrogen-bond strengths for a series of 2-hy­droxy­benzaldimine compounds range from −55 to −80 kJ mol^−1^, depending on the imine substituent (Simperler & Mikenda, 1997 ▸); and the inter­action energy for pyridine and formic acid is −46.4 kJ mol^−1^ (Fernandez-Berridi *et al.*, 2002 ▸).

## Database survey   

In 3-[1-(3-hy­droxy­benz­yl)-1*H*-benzimidazol-2-yl]phenol, the 3-hy­droxy­phenyl substituent forms a dihedral angle of 56.55 (3)° with the benzimidazole moiety (Eltayeb, Teoh, Fun *et al.*, 2009 ▸). The structure of 1-(2-hy­droxy­benz­yl)-2-(2-hy­droxy­phen­yl)-1*H*-benzimidazol-3-ium chloride displays a dihedral angle of 55.49 (9)° between the benzimidazole core and the 2-hy­droxy­phenyl substituent (Khan *et al.*, 2017 ▸). 2-(1*H*-Benzimidazol-2-yl)phenol is essentially planar and exhibits an intra­molecular hydrogen bond (Prakash *et al.*, 2014 ▸). In the hydro­chloride salt of 2-(4-hy­droxy­phen­yl)-1*H*-benzimidazole, the hy­droxy­phenyl substituent is essentially coplanar with the benzimidazole moiety (González-Padilla *et al.*, 2013 ▸). Other benzimidazole derivatives that have hy­droxy­phenyl substituents include 2-{[2-(pyridin-4-yl)-1*H*-benzimidazol-1-yl]meth­yl}phenol **(**Omer *et al.*, 2013 ▸), 2-[(1*H*-benzimidazol-1-yl)meth­yl]phenol benzene hemisolvate (Rivera *et al.*, 2014 ▸), 2-(1-phenyl-1*H*-benzimidazol-2-yl)phenol (Thiruvalluvar *et al.*, 2013 ▸), and 2-meth­oxy-6-(6-methyl-1*H*-benzimid­azol-2-yl)phenol (Eltayeb, Teoh, Quah *et al.*, 2009 ▸).

## Synthesis and crystallization   

**5,6-Dimethyl-2-(4-hy­droxy­phen­yl)-1-[(4-hy­droxy­phen­yl)meth­­yl]-1** ***H*** **-benzimidazole:** 1.96 g (14.37 mmol) of 4,5-di­meth­yl-1,2-di­amino­benzene were dissolved in 50 mL of ethanol and stirred under nitro­gen. 3.65 g (29.9 mmol) of 4-hy­droxy­benzaldehyde were dissolved in ethanol, purged with nitro­gen for 5 min., and then added dropwise to the solution. The solution was refluxed for 24 h and cooled, after which a yellow solid formed. This solid was filtered and washed with cold ethanol. 4.23 g (12.3 mmol, 85.5% yield) of the yellow solid was obtained. *R*_f_ (3:1 acetone/hexa­ne) = 0.64. ^1^H NMR (400MHz, DMSO): δ 2.23 ppm (*s*, 3H), δ 2.28 ppm (*s*, 3H), δ 5.33 ppm (*s*, 2H), δ 6.64 ppm (*d*, 2H), δ 6.78 ppm (*d*, 2H), δ 6.85 ppm (*d*, 2H), δ 7.15 ppm (*s*, 1H) δ 7.40 ppm (*s*, 1H), δ 7.50 ppm (*d*, 2H), δ 9.34 ppm (*s*, 1H), δ 9.88 ppm (*s*, 1H).

Single crystals of (1) were obtained by slow evaporation of a dilute acetone solution of the product.

## Refinement   

Crystal data, data collection and structure refinement details are summarized in Table 3 ▸. A refined extinction coefficient [0.006 (2)] was employed to calculate the correction factor applied to the structure-factor data. H atoms bonded to C were refined using a riding model with C—H = 0.95 Å for H bonded to aromatic C atoms, 0.99 Å for methyl­ene H atoms, and 0.98 Å for the methyl H atoms. *U*_iso_(H) = *kU*_eq_(C), where *k* = 1.2 for H atoms bonded to aromatic and methyl­ene C atoms and 1.5 for H atoms bonded to methyl C atoms. H atoms bonded to oxygen were refined freely, including isotropic displacement parameters.

## Hirshfeld surface, fingerprint plots, inter­action energy calculations   

Hirshfeld surfaces, fingerprint plots, and inter­action energies were calculated using *CrystalExplorer17* (Turner *et al.*, 2017 ▸), in which the C—H bond lengths were converted to normalized values based on neutron diffraction results (Allen *et al.*, 2004 ▸). Inter­action energies were calculated employing the CE-B3LYP/6-31G(*d,p*) functional/basis set combination and are corrected for basis set superposition energy (BSSE) using the counterpoise (CP) method (Boys & Bernardi, 1970 ▸). The inter­action energy is broken down as

*E*_tot_ = *k*_ele_ *E′*_ele_ + *k*_pol_ *E′*_pol_ + *k*_dis_ *E′*_dis_ + *k*_rep_ *E′*_rep_

where the *k* values are scale factors, *E′*_ele_ represents the electrostatic component, *E′*_pol_ the polarization energy, *E′*_dis_ the dispersion energy, and *E′*_rep_ the exchange-repulsion energy (Turner *et al.*, 2014 ▸; Mackenzie *et al.*, 2017 ▸).

Inter­action energy calculations were also performed on mol­ecules in the gas phase using *SPARTAN’16* (Wavefunction, 2016 ▸). DFT calculations using the M06-2X (Zhao & Truhlar, 2008 ▸) functional with a 6-31G(*d,p*) basis set were employed for the determination of inter­action energies, which were corrected for BSSE employing the CP method (Boys & Bernardi, 1970 ▸). Atomic coordinates obtained from the crystallographic analysis were used for all non-H atoms. Because bond lengths obtained for H atoms from X-ray crystallographic analyses are unreliable, the positions of the H atoms were optimized to their energy minima using the M06-2X/6-31G(*d,p*) functional/basis set combination.

## Supplementary Material

Crystal structure: contains datablock(s) global, I. DOI: 10.1107/S2056989019001270/zl2750sup1.cif

Structure factors: contains datablock(s) I. DOI: 10.1107/S2056989019001270/zl2750Isup2.hkl

Supporting information file. DOI: 10.1107/S2056989019001270/zl2750Isup3.mol

Supporting information file. DOI: 10.1107/S2056989019001270/zl2750Isup4.cml

CCDC reference: 1893078

Additional supporting information:  crystallographic information; 3D view; checkCIF report

## Figures and Tables

**Table 1:** Hydrogen-bond geometry (Å, °) *Cg*3 is ring centroid of the 2-(4-hy­droxy­phen­yl) substituent. *Cg*2 is ring centroid of the benzene ring of the benzimidazole ring system.

*D*—H⋯*A*	*D*—H	H⋯*A*	*D*⋯*A*	*D*—H⋯*A*
O1—H1⋯O4	0.81 (4)	1.96 (4)	2.748 (3)	165 (4)
O2—H2⋯N2^i^	0.93 (3)	1.83 (3)	2.757 (2)	176 (2)
C16—H16⋯*Cg*3^ii^	0.95	2.76	3.6061 (19)	149
C13—H13⋯*Cg*2^iii^	0.95	3.00	3.552 (2)	119

Symmetry codes: (i) 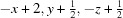; (ii) 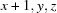; (iii) 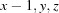.

**Table 2:** Inter­action energies calculated for (1) Energies are in kJ mol^−1^ and are corrected for BSSE.

Inter­action	*E*′_ele_	*E*′_pol_	*E*′_dis_	*E*′_rep_	*E*_tot_^1,2^	*E* ^3^
O—H⋯N	−73.0	−19.9	−20.2	82	−58.8	−39.2
O—H⋯O	−48.9	−11.0	−9.0	50.1	−36.7	−35.7
C—H⋯π	−15.1	−4.5	−90.5	56.1	−63.5	−48.5

Notes: (i) Scale factors used to determine *E*_tot_: *k*_ele_ = 1.057, *k*_pol_ = 0.740, *k*_energy-dispersive_ = 0.871, *k*_rep_ = 0.618 (Mackenzie *et al.*, 2017 ▸). See Section 7 for calculation details. (ii) Inter­action energies were calculated employing the CE-B3LYP/6–31G(*d,p*) functional/basis set combination. (iii) Inter­action energies were calculated employing the M06–2X/6–31G(*d,p*) functional/basis set combination.

**Table 3:** Experimental details

Crystal data	
Chemical formula	C_22_H_20_N_2_O_2_·2C_3_H_6_O
*M*_r_	460.55
Crystal system, space group	Monoclinic, *P*2_1_/*c*
Temperature (K)	200
*a*, *b*, *c* (Å)	5.7307 (6), 19.733 (2), 22.436 (3)
β (°)	92.400 (4)
*V* (Å^3^)	2534.9 (5)
*Z*	4
Radiation type	Mo *K*α
μ (mm^−1^)	0.08
Crystal size (mm)	0.60 × 0.30 × 0.30

Data collection	
Diffractometer	Bruker SMART X2S benchtop
Absorption correction	Multi-scan (*SADABS*; Bruker, 2015 ▸)
*T*_min_, *T*_max_	0.35, 0.98
No. of measured, independent and observed [*I* > 2σ(*I*)] reflections	26416, 4489, 3268
*R*_int_	0.093
(sin θ/λ)_max_ (Å^−1^)	0.596

Refinement	
*R*[*F* ^2^ > 2σ(*F* ^2^)], *wR*(*F* ^2^), *S*	0.058, 0.195, 1.07
No. of reflections	4489
No. of parameters	322
H-atom treatment	H atoms treated by a mixture of independent and constrained refinement
Δρ_max_, Δρ_min_ (e Å^−3^)	0.28, −0.29

Computer programs: *APEX2* and *SAINT* (Bruker, 2015 ▸), *SHELXS97* (Sheldrick, 2008 ▸), *SHELXL2014/7* (Sheldrick, 2015 ▸), *PLATON* (Spek, 2009 ▸), *Mercury* (Macrae *et al.*, 2008 ▸) and *publCIF* (Westrip, 2010 ▸).

## supplementary crystallographic information

### Crystal data

**Table:**

C_22_H_20_N_2_O_2_·2C_3_H_6_O	*F*(000) = 984
*M**_r_* = 460.55	*D*_x_ = 1.207 Mg m^−^^3^
Monoclinic, *P*2_1_/*c*	Mo *K*α radiation, λ = 0.71073 Å
*a* = 5.7307 (6) Å	Cell parameters from 7680 reflections
*b* = 19.733 (2) Å	θ = 2.3–25.0°
*c* = 22.436 (3) Å	µ = 0.08 mm^−^^1^
β = 92.400 (4)°	*T* = 200 K
*V* = 2534.9 (5) Å^3^	Prism, clear colourless
*Z* = 4	0.60 × 0.30 × 0.30 mm

### Data collection

**Table:**

Bruker SMART X2S benchtop diffractometer	4489 independent reflections
Radiation source: XOS X-beam microfocus source	3268 reflections with *I* > 2σ(*I*)
Doubly curved silicon crystal monochromator	*R*_int_ = 0.093
Detector resolution: 8.3330 pixels mm^-1^	θ_max_ = 25.1°, θ_min_ = 2.1°
ω scans	*h* = −6→6
Absorption correction: multi-scan (SADABS; Bruker, 2015)	*k* = −22→23
*T*_min_ = 0.35, *T*_max_ = 0.98	*l* = −26→26
26416 measured reflections	

### Refinement

**Table:**

Refinement on *F*^2^	Secondary atom site location: difference Fourier map
Least-squares matrix: full	Hydrogen site location: mixed
*R*[*F*^2^ > 2σ(*F*^2^)] = 0.058	H atoms treated by a mixture of independent and constrained refinement
*wR*(*F*^2^) = 0.195	*w* = 1/[σ^2^(*F*_o_^2^) + (0.124*P*)^2^] where *P* = (*F*_o_^2^ + 2*F*_c_^2^)/3
*S* = 1.07	(Δ/σ)_max_ = 0.001
4489 reflections	Δρ_max_ = 0.28 e Å^−^^3^
322 parameters	Δρ_min_ = −0.28 e Å^−^^3^
0 restraints	Extinction correction: SHELXL-2014/7 (Sheldrick 2015)
Primary atom site location: structure-invariant direct methods	Extinction coefficient: 0.006 (2)

### Special details

**Table:**

Geometry. All esds (except the esd in the dihedral angle between two l.s. planes) are estimated using the full covariance matrix. The cell esds are taken into account individually in the estimation of esds in distances, angles and torsion angles; correlations between esds in cell parameters are only used when they are defined by crystal symmetry. An approximate (isotropic) treatment of cell esds is used for estimating esds involving l.s. planes.
Refinement. Refinement of F^2^ against ALL reflections. The weighted R-factor wR and goodness of fit S are based on F^2^, conventional R-factors R are based on F, with F set to zero for negative F^2^. The threshold expression of F^2^ > 2sigma(F^2^) is used only for calculating R-factors(gt) etc. and is not relevant to the choice of reflections for refinement. R-factors based on F^2^ are statistically about twice as large as those based on F, and R- factors based on ALL data will be even larger.

### Fractional atomic coordinates and isotropic or equivalent isotropic displacement parameters (Å^2^)

**Table:**

	*x*	*y*	*z*	*U*_iso_*/*U*_eq_	
O1	0.0984 (3)	0.33911 (10)	0.39918 (10)	0.0665 (6)	
H1	0.154 (7)	0.3632 (18)	0.425 (2)	0.110 (14)*	
O2	1.3625 (3)	0.65683 (6)	0.31799 (8)	0.0513 (5)	
H2	1.304 (5)	0.7006 (14)	0.3166 (12)	0.066 (7)*	
O3	0.3839 (4)	0.58204 (10)	0.11484 (12)	0.0973 (8)	
O4	0.2047 (4)	0.42019 (11)	0.49612 (10)	0.0795 (6)	
N1	0.8301 (3)	0.40174 (7)	0.19126 (8)	0.0357 (5)	
N2	0.7935 (3)	0.28900 (7)	0.18639 (8)	0.0378 (5)	
C1	0.9855 (3)	0.37992 (8)	0.14968 (10)	0.0367 (5)	
C2	0.9600 (3)	0.30963 (8)	0.14685 (10)	0.0379 (5)	
C3	1.1005 (4)	0.27223 (9)	0.11001 (11)	0.0459 (6)	
H3	1.0836	0.2244	0.1076	0.055*	
C4	1.2651 (4)	0.30477 (11)	0.07677 (11)	0.0487 (6)	
C5	1.2878 (4)	0.37676 (10)	0.07961 (11)	0.0469 (6)	
C6	1.1473 (4)	0.41409 (9)	0.11616 (10)	0.0435 (6)	
H6	1.161	0.462	0.1183	0.052*	
C7	0.7220 (3)	0.34487 (8)	0.21232 (10)	0.0341 (5)	
C8	0.5588 (3)	0.34613 (8)	0.26094 (10)	0.0362 (5)	
C9	0.6028 (4)	0.38479 (9)	0.31208 (11)	0.0405 (6)	
H9	0.7375	0.4129	0.3149	0.049*	
C10	0.4532 (4)	0.38297 (9)	0.35902 (11)	0.0446 (6)	
H10	0.4866	0.4093	0.3938	0.054*	
C11	0.2546 (4)	0.34270 (10)	0.35521 (11)	0.0444 (6)	
C12	0.2085 (4)	0.30419 (10)	0.30451 (11)	0.0474 (6)	
H12	0.0719	0.2769	0.3015	0.057*	
C13	0.3607 (4)	0.30533 (9)	0.25829 (10)	0.0409 (6)	
H13	0.3294	0.2778	0.2242	0.049*	
C14	0.7491 (3)	0.47223 (8)	0.19678 (10)	0.0387 (5)	
H14A	0.5982	0.4719	0.2167	0.046*	
H14B	0.7199	0.4909	0.1562	0.046*	
C15	0.9149 (3)	0.51921 (8)	0.23098 (9)	0.0326 (5)	
C16	1.1243 (3)	0.49868 (8)	0.25815 (10)	0.0367 (5)	
H16	1.1679	0.4523	0.257	0.044*	
C17	1.2716 (3)	0.54515 (8)	0.28710 (10)	0.0378 (5)	
H17	1.4148	0.5302	0.3055	0.045*	
C18	1.2115 (4)	0.61319 (8)	0.28943 (10)	0.0359 (5)	
C19	0.9987 (4)	0.63368 (8)	0.26372 (10)	0.0414 (6)	
H19	0.953	0.6798	0.266	0.05*	
C20	0.8527 (3)	0.58733 (9)	0.23476 (10)	0.0405 (6)	
H20	0.7079	0.6021	0.2172	0.049*	
C21	0.2570 (5)	0.62914 (14)	0.10570 (13)	0.0626 (7)	
C22	0.0308 (7)	0.6214 (2)	0.0731 (2)	0.1300 (18)	
H22A	0.0203	0.5759	0.0555	0.195*	
H22B	0.0169	0.6554	0.0413	0.195*	
H22C	−0.0957	0.6276	0.1006	0.195*	
C23	0.3276 (7)	0.69718 (15)	0.1289 (2)	0.1050 (13)	
H23A	0.2746	0.7026	0.1696	0.158*	
H23B	0.2566	0.7324	0.1032	0.158*	
H23C	0.4981	0.7013	0.1292	0.158*	
C24	0.0807 (5)	0.42468 (12)	0.53839 (13)	0.0580 (7)	
C25	−0.1601 (5)	0.39762 (17)	0.53648 (16)	0.0822 (10)	
H25A	−0.2715	0.4351	0.5316	0.123*	
H25B	−0.1877	0.3736	0.5738	0.123*	
H25C	−0.1808	0.3662	0.5028	0.123*	
C26	0.1701 (7)	0.45779 (19)	0.59377 (15)	0.0916 (11)	
H26A	0.3078	0.485	0.5853	0.137*	
H26B	0.2131	0.4231	0.6235	0.137*	
H26C	0.0488	0.4872	0.6092	0.137*	
C41	1.4225 (5)	0.26394 (14)	0.03844 (15)	0.0718 (8)	
H41A	1.3985	0.2155	0.0458	0.108*	
H41B	1.5858	0.2757	0.0483	0.108*	
H41C	1.3858	0.274	−0.0037	0.108*	
C51	1.4674 (5)	0.41265 (14)	0.04431 (13)	0.0649 (7)	
H51A	1.4655	0.4612	0.0537	0.097*	
H51B	1.4314	0.4062	0.0016	0.097*	
H51C	1.6224	0.394	0.0546	0.097*	

### Atomic displacement parameters (Å^2^)

**Table:**

	*U*^11^	*U*^22^	*U*^33^	*U*^12^	*U*^13^	*U*^23^
O1	0.0602 (12)	0.0842 (12)	0.0556 (13)	−0.0163 (9)	0.0101 (10)	−0.0131 (10)
O2	0.0532 (10)	0.0285 (7)	0.0705 (12)	−0.0013 (6)	−0.0172 (9)	−0.0070 (6)
O3	0.1049 (17)	0.0705 (11)	0.114 (2)	0.0236 (12)	−0.0186 (15)	−0.0043 (13)
O4	0.0804 (14)	0.0976 (14)	0.0612 (15)	−0.0162 (11)	0.0109 (11)	−0.0186 (12)
N1	0.0392 (9)	0.0242 (7)	0.0432 (11)	0.0010 (6)	−0.0033 (8)	−0.0035 (7)
N2	0.0433 (10)	0.0270 (7)	0.0429 (11)	0.0003 (6)	−0.0030 (8)	−0.0021 (7)
C1	0.0390 (12)	0.0320 (8)	0.0386 (12)	0.0010 (8)	−0.0061 (10)	−0.0018 (8)
C2	0.0418 (12)	0.0298 (8)	0.0411 (13)	0.0024 (8)	−0.0089 (10)	−0.0020 (8)
C3	0.0534 (14)	0.0358 (9)	0.0476 (14)	0.0036 (9)	−0.0057 (11)	−0.0073 (9)
C4	0.0509 (13)	0.0519 (11)	0.0427 (14)	0.0081 (10)	−0.0043 (11)	−0.0119 (10)
C5	0.0464 (13)	0.0519 (11)	0.0416 (14)	−0.0005 (10)	−0.0055 (11)	−0.0007 (10)
C6	0.0481 (13)	0.0375 (9)	0.0443 (14)	−0.0036 (9)	−0.0049 (11)	0.0016 (9)
C7	0.0342 (11)	0.0282 (8)	0.0394 (12)	−0.0011 (7)	−0.0068 (9)	−0.0003 (8)
C8	0.0361 (11)	0.0286 (8)	0.0430 (13)	0.0008 (7)	−0.0076 (10)	0.0001 (8)
C9	0.0402 (12)	0.0356 (9)	0.0451 (14)	−0.0048 (8)	−0.0056 (10)	−0.0027 (9)
C10	0.0483 (13)	0.0413 (9)	0.0434 (14)	−0.0034 (9)	−0.0079 (11)	−0.0086 (9)
C11	0.0416 (12)	0.0499 (11)	0.0416 (13)	−0.0003 (9)	−0.0005 (10)	−0.0004 (10)
C12	0.0449 (13)	0.0449 (10)	0.0520 (15)	−0.0122 (9)	−0.0038 (12)	−0.0032 (10)
C13	0.0418 (12)	0.0354 (9)	0.0447 (13)	−0.0035 (8)	−0.0076 (10)	−0.0060 (9)
C14	0.0399 (11)	0.0242 (8)	0.0513 (14)	0.0044 (7)	−0.0073 (9)	−0.0029 (8)
C15	0.0370 (11)	0.0250 (8)	0.0358 (12)	0.0011 (7)	−0.0001 (9)	0.0004 (8)
C16	0.0405 (11)	0.0230 (7)	0.0463 (13)	0.0053 (7)	0.0001 (9)	−0.0016 (8)
C17	0.0364 (11)	0.0312 (8)	0.0455 (13)	0.0056 (7)	−0.0048 (9)	−0.0025 (8)
C18	0.0422 (11)	0.0252 (7)	0.0400 (12)	−0.0016 (7)	−0.0013 (9)	−0.0004 (8)
C19	0.0492 (12)	0.0216 (7)	0.0530 (14)	0.0060 (8)	−0.0034 (10)	−0.0011 (8)
C20	0.0400 (11)	0.0290 (8)	0.0518 (14)	0.0062 (7)	−0.0072 (10)	0.0025 (8)
C21	0.0641 (17)	0.0717 (15)	0.0526 (17)	0.0047 (13)	0.0095 (13)	0.0064 (13)
C22	0.094 (3)	0.183 (4)	0.110 (4)	−0.017 (3)	−0.031 (3)	0.046 (3)
C23	0.124 (3)	0.0691 (17)	0.124 (4)	0.0031 (19)	0.026 (3)	−0.017 (2)
C24	0.0678 (17)	0.0531 (12)	0.0526 (17)	−0.0043 (12)	−0.0010 (13)	0.0046 (12)
C25	0.071 (2)	0.100 (2)	0.075 (2)	−0.0180 (17)	−0.0023 (16)	0.0165 (18)
C26	0.112 (3)	0.108 (2)	0.056 (2)	−0.038 (2)	0.0110 (18)	−0.0129 (18)
C41	0.0707 (18)	0.0746 (16)	0.071 (2)	0.0101 (14)	0.0105 (15)	−0.0199 (15)
C51	0.0612 (16)	0.0765 (15)	0.0573 (18)	−0.0092 (13)	0.0066 (13)	−0.0028 (14)

### Geometric parameters (Å, º)

**Table:**

O1—C11	1.361 (3)	C14—H14B	0.99
O1—H1	0.81 (4)	C15—C16	1.384 (2)
O2—C18	1.362 (2)	C15—C20	1.394 (2)
O2—H2	0.93 (3)	C16—C17	1.389 (2)
O3—C21	1.192 (3)	C16—H16	0.95
O4—C24	1.212 (3)	C17—C18	1.388 (2)
N1—C7	1.375 (2)	C17—H17	0.95
N1—C1	1.385 (3)	C18—C19	1.387 (3)
N1—C14	1.473 (2)	C19—C20	1.383 (3)
N2—C7	1.320 (2)	C19—H19	0.95
N2—C2	1.391 (3)	C20—H20	0.95
C1—C6	1.392 (3)	C21—C22	1.470 (4)
C1—C2	1.396 (2)	C21—C23	1.490 (4)
C2—C3	1.390 (3)	C22—H22A	0.98
C3—C4	1.385 (3)	C22—H22B	0.98
C3—H3	0.95	C22—H22C	0.98
C4—C5	1.428 (3)	C23—H23A	0.98
C4—C41	1.506 (4)	C23—H23B	0.98
C5—C6	1.385 (3)	C23—H23C	0.98
C5—C51	1.502 (4)	C24—C26	1.477 (4)
C6—H6	0.95	C24—C25	1.479 (4)
C7—C8	1.466 (3)	C25—H25A	0.98
C8—C13	1.391 (3)	C25—H25B	0.98
C8—C9	1.392 (3)	C25—H25C	0.98
C9—C10	1.386 (3)	C26—H26A	0.98
C9—H9	0.95	C26—H26B	0.98
C10—C11	1.388 (3)	C26—H26C	0.98
C10—H10	0.95	C41—H41A	0.98
C11—C12	1.384 (3)	C41—H41B	0.98
C12—C13	1.383 (3)	C41—H41C	0.98
C12—H12	0.95	C51—H51A	0.98
C13—H13	0.95	C51—H51B	0.98
C14—C15	1.513 (2)	C51—H51C	0.98
C14—H14A	0.99		
			
C11—O1—H1	104 (3)	C17—C16—H16	119.6
C18—O2—H2	110.6 (17)	C18—C17—C16	120.63 (16)
C7—N1—C1	106.82 (15)	C18—C17—H17	119.7
C7—N1—C14	126.42 (18)	C16—C17—H17	119.7
C1—N1—C14	124.31 (17)	O2—C18—C19	122.80 (15)
C7—N2—C2	105.70 (15)	O2—C18—C17	118.41 (16)
N1—C1—C6	132.44 (16)	C19—C18—C17	118.78 (16)
N1—C1—C2	105.69 (19)	C20—C19—C18	120.44 (15)
C6—C1—C2	121.8 (2)	C20—C19—H19	119.8
C3—C2—N2	130.84 (16)	C18—C19—H19	119.8
C3—C2—C1	119.5 (2)	C19—C20—C15	121.00 (16)
N2—C2—C1	109.58 (19)	C19—C20—H20	119.5
C4—C3—C2	119.92 (18)	C15—C20—H20	119.5
C4—C3—H3	120.0	O3—C21—C22	121.5 (3)
C2—C3—H3	120.0	O3—C21—C23	119.2 (3)
C3—C4—C5	120.0 (2)	C22—C21—C23	119.3 (3)
C3—C4—C41	119.8 (2)	C21—C22—H22A	109.5
C5—C4—C41	120.2 (2)	C21—C22—H22B	109.5
C6—C5—C4	120.1 (2)	H22A—C22—H22B	109.5
C6—C5—C51	119.3 (2)	C21—C22—H22C	109.5
C4—C5—C51	120.6 (2)	H22A—C22—H22C	109.5
C5—C6—C1	118.62 (18)	H22B—C22—H22C	109.5
C5—C6—H6	120.7	C21—C23—H23A	109.5
C1—C6—H6	120.7	C21—C23—H23B	109.5
N2—C7—N1	112.2 (2)	H23A—C23—H23B	109.5
N2—C7—C8	124.15 (16)	C21—C23—H23C	109.5
N1—C7—C8	123.52 (16)	H23A—C23—H23C	109.5
C13—C8—C9	118.1 (2)	H23B—C23—H23C	109.5
C13—C8—C7	120.22 (18)	O4—C24—C26	119.8 (3)
C9—C8—C7	121.54 (17)	O4—C24—C25	121.9 (3)
C10—C9—C8	121.03 (18)	C26—C24—C25	118.4 (3)
C10—C9—H9	119.5	C24—C25—H25A	109.5
C8—C9—H9	119.5	C24—C25—H25B	109.5
C9—C10—C11	120.0 (2)	H25A—C25—H25B	109.5
C9—C10—H10	120.0	C24—C25—H25C	109.5
C11—C10—H10	120.0	H25A—C25—H25C	109.5
O1—C11—C12	117.3 (2)	H25B—C25—H25C	109.5
O1—C11—C10	123.2 (2)	C24—C26—H26A	109.5
C12—C11—C10	119.5 (2)	C24—C26—H26B	109.5
C13—C12—C11	120.17 (19)	H26A—C26—H26B	109.5
C13—C12—H12	119.9	C24—C26—H26C	109.5
C11—C12—H12	119.9	H26A—C26—H26C	109.5
C12—C13—C8	121.11 (19)	H26B—C26—H26C	109.5
C12—C13—H13	119.4	C4—C41—H41A	109.5
C8—C13—H13	119.4	C4—C41—H41B	109.5
N1—C14—C15	115.33 (14)	H41A—C41—H41B	109.5
N1—C14—H14A	108.4	C4—C41—H41C	109.5
C15—C14—H14A	108.4	H41A—C41—H41C	109.5
N1—C14—H14B	108.4	H41B—C41—H41C	109.5
C15—C14—H14B	108.4	C5—C51—H51A	109.5
H14A—C14—H14B	107.5	C5—C51—H51B	109.5
C16—C15—C20	118.32 (15)	H51A—C51—H51B	109.5
C16—C15—C14	123.96 (14)	C5—C51—H51C	109.5
C20—C15—C14	117.72 (15)	H51A—C51—H51C	109.5
C15—C16—C17	120.78 (15)	H51B—C51—H51C	109.5
C15—C16—H16	119.6		
			
C7—N1—C1—C6	−176.90 (19)	N2—C7—C8—C13	−43.7 (2)
C14—N1—C1—C6	19.9 (3)	N1—C7—C8—C13	140.81 (17)
C7—N1—C1—C2	1.05 (18)	N2—C7—C8—C9	132.74 (18)
C14—N1—C1—C2	−162.14 (15)	N1—C7—C8—C9	−42.8 (2)
C7—N2—C2—C3	176.53 (19)	C13—C8—C9—C10	−0.2 (3)
C7—N2—C2—C1	−0.28 (19)	C7—C8—C9—C10	−176.75 (16)
N1—C1—C2—C3	−177.72 (16)	C8—C9—C10—C11	−0.7 (3)
C6—C1—C2—C3	0.5 (3)	C9—C10—C11—O1	−179.10 (19)
N1—C1—C2—N2	−0.49 (19)	C9—C10—C11—C12	0.5 (3)
C6—C1—C2—N2	177.73 (16)	O1—C11—C12—C13	−179.72 (19)
N2—C2—C3—C4	−176.16 (18)	C10—C11—C12—C13	0.7 (3)
C1—C2—C3—C4	0.4 (3)	C11—C12—C13—C8	−1.7 (3)
C2—C3—C4—C5	−1.0 (3)	C9—C8—C13—C12	1.4 (3)
C2—C3—C4—C41	178.0 (2)	C7—C8—C13—C12	177.96 (17)
C3—C4—C5—C6	0.8 (3)	C7—N1—C14—C15	119.1 (2)
C41—C4—C5—C6	−178.2 (2)	C1—N1—C14—C15	−81.0 (2)
C3—C4—C5—C51	179.3 (2)	N1—C14—C15—C16	−2.9 (3)
C41—C4—C5—C51	0.3 (3)	N1—C14—C15—C20	176.6 (2)
C4—C5—C6—C1	0.0 (3)	C20—C15—C16—C17	−1.7 (3)
C51—C5—C6—C1	−178.48 (19)	C14—C15—C16—C17	177.8 (2)
N1—C1—C6—C5	176.97 (19)	C15—C16—C17—C18	0.1 (4)
C2—C1—C6—C5	−0.7 (3)	C16—C17—C18—O2	−179.6 (2)
C2—N2—C7—N1	0.98 (19)	C16—C17—C18—C19	1.7 (4)
C2—N2—C7—C8	−174.96 (16)	O2—C18—C19—C20	179.4 (2)
C1—N1—C7—N2	−1.31 (19)	C17—C18—C19—C20	−1.9 (4)
C14—N1—C7—N2	161.42 (17)	C18—C19—C20—C15	0.3 (4)
C1—N1—C7—C8	174.66 (16)	C16—C15—C20—C19	1.5 (4)
C14—N1—C7—C8	−22.6 (3)	C14—C15—C20—C19	−178.0 (2)

### Hydrogen-bond geometry (Å, º)

*Cg*3 is ring centroid of the 2-(4-hydroxyphenyl) substituent. *Cg*2 is ring centroid of the benzene ring of the benzimidazole ring system.

**Table:**

*D*—H···*A*	*D*—H	H···*A*	*D*···*A*	*D*—H···*A*
O1—H1···O4	0.81 (4)	1.96 (4)	2.748 (3)	165 (4)
O2—H2···N2^i^	0.93 (3)	1.83 (3)	2.757 (2)	176 (2)
C16—H16···*Cg*3^ii^	0.95	2.76	3.6061 (19)	149
C13—H13···*Cg*2^iii^	0.95	3.00	3.552 (2)	119

Symmetry codes: (i) −*x*+2, *y*+1/2, −*z*+1/2; (ii) *x*+1, *y*, *z*; (iii) *x*−1, *y*, *z*.
